# 14-3-3*η* Protein as a Potential Biomarker in Juvenile Idiopathic Arthritis

**DOI:** 10.3390/pediatric13010008

**Published:** 2021-01-25

**Authors:** Austin Dalrymple, Paul Tuttle, Lance Feller, Olga Zhukov, Robert Lagier, Joanna Popov, Stanley Naides, Terry Moore

**Affiliations:** 1 Division of Adult & Pediatric Rheumatology, Saint Louis University School of Medicine & SSM Health Cardinal Glennon Children’s Hospital, Saint Louis, MO 63104, USA; paultuttleiv@gmail.com (P.T.); lfeller@gmail.com (L.F.); terry.moore@health.slu.edu (T.M.); 2 Quest Diagnostics Nichols Institute, San Juan Capistrano, CA 92675, USA; Olga.S.Zhukov@questdiagnostics.com (O.Z.); Robert.J.Lagier@questdiagnostics.com (R.L.); Joanna.M.Popov@questdiagnostics.com (J.P.); stanley.naides@gmail.com (S.N.)

**Keywords:** juvenile idiopathic arthritis, 14-3-3 proteins, biomarkers

## Abstract

The 14-3-3***η*** (eta) protein was evaluated as a biomarker in a cohort of patients with juvenile idiopathic arthritis (JIA), as well as disease- and healthy-controls, to determine its potential clinical utility. In this case-control study, levels of 14-3-3***η*** protein were evaluated in archival specimens from patients with JIA, systemic lupus erythematosus (SLE), and rheumatoid arthritis (RA), as well as healthy pediatric controls. Just over 200 patients were evaluated, using specimens banked between 1990 and 2011. Comparisons were made to complete blood cell count (CBC), erythrocyte sedimentation rate (ESR), C-reactive protein (CRP), rheumatoid factor (RF), anti-cyclic citrullinated peptide (anti-CCP) antibodies, and anti-nuclear antibody (ANA) positivity. 14-3-3***η*** at levels 0.2 ng/mL or higher was considered positive. Fisher’s exact tests, odds ratios, 95% confidence intervals, and p-values were reported. 14-3-3***η*** positivity was seen in all included JIA subtypes. The rate of positivity was the highest in RF-positive (pos) polyarticular JIA. In the disease and healthy controls, lower rates of positivity were observed. The frequency of 14-3-3***η*** positivity among RF-positive and RF-negative (neg) polyarticular JIA patients, especially at values ≥0.5 ng/mL (associated with poor outcomes in adults), was also highest. Several JIA patients with 14-3-3***η*** positivity developed RF and anti-CCP positivity later in their disease. Significant levels of 14-3-3***η*** can be found in approximately 30% of RF-pos and RF-neg patients with polyarticular JIA. This protein may represent a new biomarker for polyarticular JIA, particularly RF-neg polyarticular JIA.

## 1. Introduction

JIA is the most common rheumatic disease of childhood, yet disease-specific diagnostic biomarkers are not available [1]. While various biomarkers have been evaluated in JIA, including rheumatoid factor (RF), anti-cyclic citrullinated peptide (anti-CCP) antibodies, anti-nuclear antibodies (ANA), and anti-carbamylated proteins (anti-CarP), none provide robust diagnostic utility [2,3,4,5,6,7,8]. A biomarker specific to JIA disease activity could minimize diagnostic delay and complications, and ultimately improve outcomes. 

The 14-3-3***η*** protein has been evaluated for diagnostic potential in adult inflammatory arthritides, but its utility in juvenile idiopathic arthritis (JIA) is not known. 14-3-3 proteins are chaperonins found in all eukaryotic cells, and multiple isoforms are involved in several intracellular functions. Our prior investigations of 14-3-3***η*** revealed positivity in a small JIA cohort [9,10,11]. Other work has implicated the ***η*** isoform, found in synovium, as having diagnostic potential in inflammatory arthritides [9,12]. Elevated serum 14-3-3***η*** improves diagnostic sensitivity in adult RA when combined with RF and anti-CCP, and may play a role in upregulating proinflammatory cytokines in the RA joint [13,14]. Here we evaluated a larger cohort of patients with JIA, as well as controls, to determine the clinical significance of 14-3-3***η*** in JIA.

## 2. Experimental Section

In this case-control study, 14-3-3***η*** protein was measured in archived sera from children with JIA, specifically rheumatoid factor (RF)-positive (pos) polyarticular, RF-negative (neg) polyarticular, oligoarticular, and systemic-onset (SO) subtypes. Controls included adults with RA and systemic lupus erythematosus (SLE), and healthy children. Subjects were classified by American College of Rheumatology and International League of Associations for Rheumatology criteria.

Archived specimens were collected and banked at Saint Louis University School of Medicine between 1990 and 2011. 14-3-3***η*** evaluations were performed at the Quest Diagnostics Nichols Institute (San Juan Capistrano, CA, USA) in a blinded fashion using an enzyme-linked immunosorbent assay (ELISA) [13].

Data analysis was conducted in 2017–2018. Patient sex, age, and diagnosis were obtained by chart extraction, as were values for complete blood cell count (CBC), erythrocyte sedimentation rate (ESR), C-reactive protein (CRP), complement C3 (C3), RF, anti-CCP, and ANA. Disease activity scores were not available.

A 14-3-3***η*** level at ≥0.2 ng/mL was considered positive, based on adult data, as pediatric reference ranges have not been established [13]. Values of ≥0.5 ng/mL were also analyzed, since values of ≥0.5 ng/mL are a poor prognostic indicator in adults [12].

Fisher’s exact tests were used to evaluate association of JIA diagnosis and sex with 14-3-3***η*** positivity. Odds ratios (ORs), 95% confidence intervals (CIs), and *p*-values (*p*) were reported. Spearman’s rank correlation was used to assess associations between 14-3-3***η*** values and age, along with other laboratory values. Cochran-Armitage test for trend, Welch Two Sample t-test, ANOVA, and Tukey’s Honest Significant Difference were used to complete sub-analysis of age, RF, and 14-3-3***η*** (supplemental materials). Statistical calculations were made in R using R Core Team (2017), R Foundation for Statistical Computing, Vienna, Austria.

This study was approved by the Institutional Review Board of Saint Louis University School of Medicine (#3017 Immune Complexes in Juvenile Idiopathic Arthritis and other Connective Tissue Diseases). All study procedures were performed in accordance with the ethical standards of this board as well as the Declaration of Helsinki.

## 3. Results

Demographic characteristics are displayed in Table 1, and results of the 14-3-3***η*** analyses are in Table 2. The highest level of positivity was noted in polyarticular JIA, the lowest in healthy controls.

Comparisons of 14-3-3***η*** levels between disease groups and controls were made for both 0.2 ng/mL and 0.5 ng/mL thresholds (Table 3 and Table 4). The odds of 14-3-3***η*** ≥ 0.5 ng/mL being linked to polyarticular JIA were 2.9 [1.0,9.0] fold greater than observed in adult SLE controls (*p* = 0.037) (Table 4). Although no other statistically significant associations were noted, children with JIA (both RF-pos and -neg) also had higher odds of having a 14-3-3***η*** level ≥0.5 ng/mL than healthy controls (OR 7.1 [1.0,319], *p* = 0.056).

Positive proportions of 14-3-3*η* were greater in polyarticular JIA patients than for all other groups, regardless of threshold (excepting RA ≥ 0.2 ng/mL). Despite this trend in association, not all comparisons achieved levels of significance.

**Figure 1:**
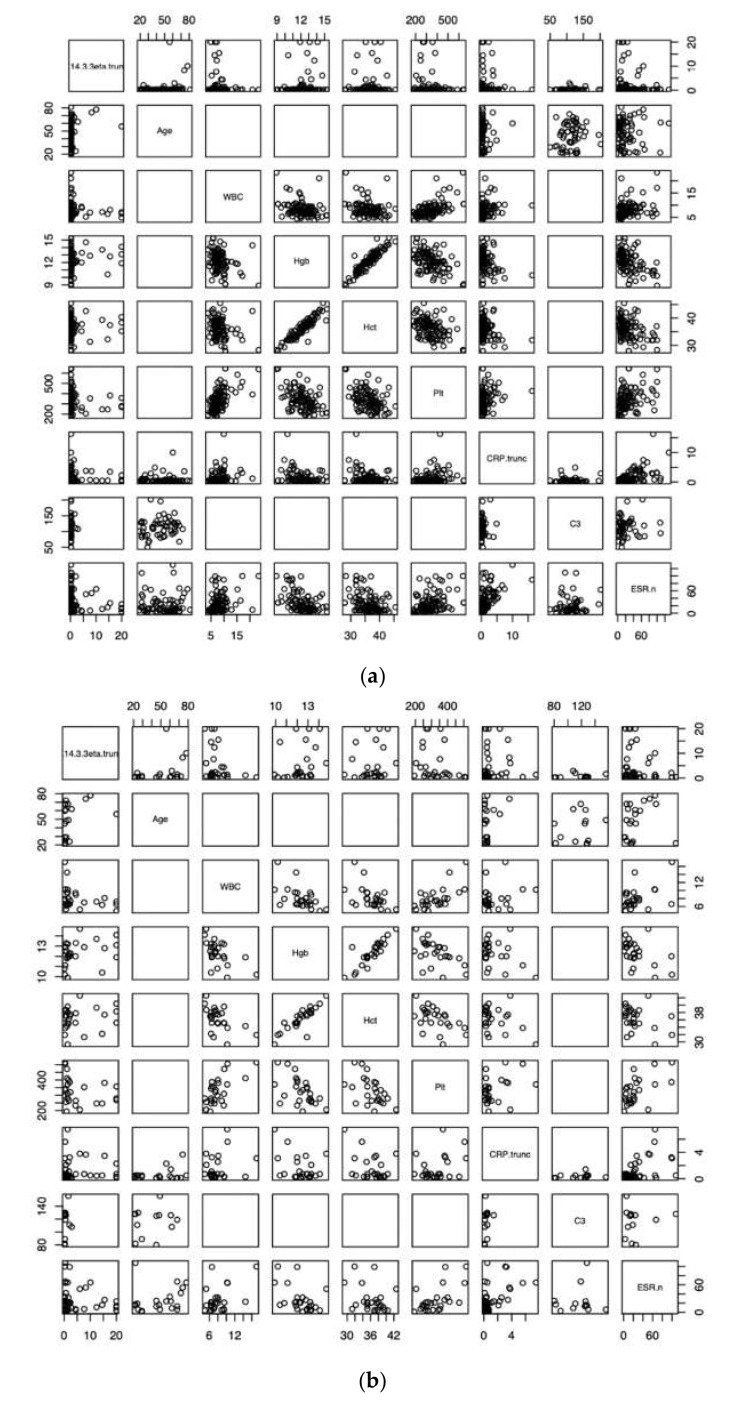
Correlation plots for all 14-3-3***η*** values (**a**) and positive 14-3-3***η*** values (**b**) vs. age, WBC, Hgb, Hct, Plt, CRP, C3, and ESR.

There was no correlation observed between 14-3-3***η*** values or 14-3-3***η*** positivity and age, white blood cell count, hemoglobin, hematocrit, platelet count, CRP, C3, or ESR, but full lab datasets were not available for all patients (Table 5 and Table 6, Figure 1).

Additional analyses revealed that older subjects appeared to have higher proportion of RF positivity, but it did not appear to be significant. ANOVA revealed that among 14-3-3***η***-pos subjects there was a difference in age based on RF status (*p* = 0.02). Further, a multiple comparison analysis suggested that the 14-3-3***η***-pos/RF-pos subjects were on average 8 years older than the 14-3-3***η***-neg/RF-neg subjects (*p* = 0.04). RF-pos subjects were approximately 10 years older than RF-neg subjects (*p* = 0.01), whereas 14-3-3***η*** was not associated with age when controlling for RF status (*p* = 0.06). Details of these analyses are available in the supplemental materials.

## 4. Discussion

In this study, we demonstrated 14-3-3***η*** positivity in JIA subgroups and disease- and healthy-controls. Adult RA was chosen as a control group because of its known association of 14-3-3***η*** positivity, and adult SLE because the positivity rate is low. The highest frequency of 14-3-3***η*** positivity (about 30%) was noted in the polyarticular JIA groups. There was a statistical difference between polyarticular JIA groups vs adult SLE controls, indicating an association between 14-3-3***η*** and polyarticular JIA. Several other trends were noted in the polyarticular JIA groups vs controls, which suggests further association with 14-3-3***η***. 

While the ultimate clinical significance of 14-3-3***η*** in polyarticular JIA patients remains unclear, elevated 14-3-3***η*** levels could indicate higher risk of aggressive disease, as do RF and anti-CCP antibodies [12,13,14]. Since 14-3-3***η*** appears to be associated with erosive disease in adult RA and psoriatic arthritis, it is worth investigating a similar association in JIA. We did not evaluate for joint erosion in this study but did see some association between 14-3-3***η*** and erosions in JIA patients in our previous cohort [10]. 

We observed 14-3-3***η*** positivity (≥0.5 ng/mL) rates of 28% in RF-pos polyarticular JIA, 28% in RF-neg polyarticular, and 28% in combined RF-pos and-neg vs 5% in healthy controls. Despite not achieving statistical significance, the result could indicate a possible association of 14-3-3***η*** with polyarticular JIA. 

Interestingly, five of eight RF-neg polyarticular patients who were 14-3-3***η*** positive subsequently developed RF and anti-CCP antibody positivity. The SO JIA patient positive for 14-3-3***η*** also developed RF later. These examples suggest that 14-3-3***η*** develops in some JIA patients prior to RF or anti-CCP and so may portend future disease. 

Limitations of this study include small sample sizes, which limited the statistical significance of our comparisons. Controls were limited based on availability of banked serum for healthy children, and adults with SLE and RA. Another limitation was due to retrospective collection of laboratory data sets, which were occasionally incomplete. Further, we did not evaluate treatment prior to blood sampling. 14-3-3***η*** has been shown to be a modifiable biomarker with treatment in adult rheumatoid arthritis [12]. Treatment may have normalized 14-3-3***η*** in some cases. 

14-3-3***η*** protein levels ≥0.5 ng/mL can be found in almost 30% of RF-pos and RF-neg polyarticular JIA patients. It appears to be present less often in other JIA subtypes and in controls. It may represent a novel biomarker for polyarticular JIA patients, with evidence suggesting that it may predict the seroconversion to RF or anti-CCP positivity. Larger longitudinal studies are required to more fully define the utility of measuring 14-3-3***η*** in children with JIA.

## Figures and Tables

**Table 1:** Demographic data by patient group.

Group	Female/Male, N	Average Age * (SD)
RF+ polyarticular	25/4	15 (+/−8)
RF- polyarticular	23/6	12 (+/−7)
Oligoarticular	32/2	9 (+/−7)
Systemic Onset	8/4	9 (+/−5)
Adult SLE	53/7	46 (+/−15)
Adult RA	11/8	55 (+/−17)
Healthy Controls	17/3	7 (+/−5)

RF = rheumatoid factor, SLE = systemic lupus erythematosus, RA = rheumatoid arthritis, SD = standard deviation.* in years at time of banked serum draw.

**Table 2:** Values of 14-3-3***η*** by group.

Group	≥0.2 ng/mL (% [95% CI])	≥0.5 ng/mL (% [95% CI])	Neg	N
RF+ polyarticular	10 (34 [18,54])	8 (28 [13,47])	11	29
RF- polyarticular	9 (31 [15,51])	8 (28 [13,47])	12	29
Oligoarticular	6 (18 [7,34])	5 (15 [5,31])	23	34
Systemic Onset	2 (17 [2,48])	1 (8 [0,38])	9	12
Disease Controls				
SLE	14 (23 [13,36])	7 (12 [5,23])	39	60
RA	7 (37 [16,62])	5 (26 [9,51])	7	19
Healthy Controls	3 (15 [3,38])	1 (5 [0,25])	16	20

RF = rheumatoid factor, SLE = systemic lupus erythematosus, RA = rheumatoid arthritis, CI = confidence interval, Neg = negative (<0.2) for 14-3-3***η***.

**Table 3:** Two-Group comparisons of 14-3-3***η*** test results using the 0.2 ng/mL cutoff.

Test Group	Reference Group	OR (95% CI)	*p* Value
RF+ polyarticular	Systemic Onset	2.6 (0.4,28.7)	0.452
	Oligoarticular	2.4 (0.7,9.6)	0.154
	Adult SLE	1.7 (0.6,5.0)	0.312
	Adult RA	0.9 (0.2,3.6)	1.000
	Healthy Controls	2.9 (0.6,19.3)	0.191
RF- polyarticular	Systemic Onset	2.2 (0.4,24.8)	0.457
	Oligoarticular	2.1 (0.6,8.3)	0.247
	Adult SLE	1.5 (0.5,4.4)	0.450
	Adult RA	0.8 (0.2,3.2)	0.759
	Healthy Controls	2.5 (0.5,16.7)	0.313
RF all polyarticular	Systemic Onset	2.4 (0.4,24.8)	0.325
	Oligoarticular	2.3 (0.7,7.8)	0.148
	Adult SLE	1.6 (0.7,3.9)	0.307
	Adult RA	0.8 (0.3,2.9)	0.784
	Healthy Controls	2.7 (0.7,16.3)	0.158

RF = rheumatoid factor, SLE = systemic lupus erythematosus, RA = rheumatoid arthritis, OR = odds ratio, CI = confidence interval.

**Table 4:** Two-Group comparisons of 14-3-3***η*** test results using the 0.5 ng/mL cutoff.

Test Group	Reference Group	OR (95% CI)	*p* Value
RF+ polyarticular	Systemic Onset	4.1 (0.4,202.4)	0.240
	Oligoarticular	2.2 (0.5,9.8)	0.230
	Adult SLE	2.8 (0.8,10.5)	0.075
	Adult RA	1.1 (0.2,5.0)	1.000
	Healthy Controls	7.0 (0.8,337.1)	0.064
RF- polyarticular	Systemic Onset	4.1 (0.4,202.4)	0.240
	Oligoarticular	2.2 (0.5,9.8)	0.230
	Adult SLE	2.8 (0.8,10.5)	0.075
	Adult RA	1.1 (0.2,5.0)	1.000
	Healthy Controls	7.0 (0.8,337.1)	0.064
RF all polyarticular	Systemic Onset	4.1 (0.5,191.3)	0.269
	Oligoarticular	2.2 (0.7,8.5)	0.202
	Adult SLE	2.9 (1.0,9.0)	0.037
	Adult RA	1.1 (0.3,4.4)	1.000
	Healthy Controls	7.1 (1.0,318.8)	0.056

RF = rheumatoid factor, SLE = systemic lupus erythematosus, RA = rheumatoid arthritis, OR = odds ratio, CI = confidence interval.

**Table 5:** Spearman’s Rank Correlation (ρ) for All 14-3-3***η***.

	14-3-3*η*	Age	WBC	Hgb	Hct	Plt	CRP	C3	ESR
14-3-3***η***	1	0.05 (*p* = 0.5)	−0.10 (0.3)	0.13 (0.2)	0.06 (0.6)	−0.10 (0.3)	0.02 (0.8)	0.11 (0.4)	0.04 (0.6)
Age		1	−0.41	0.38	0.44	−0.46	−0.37	0.15	−0.20
WBC			1	−0.30	−0.28	0.55	0.26	NA	0.30
Hgb				1	0.92	−0.44	−0.32	NA	−0.46
Hct					1	−0.43	−0.32	NA	−0.38
Plt						1	0.31	NA	0.41
CRP							1	0.09	0.59
C3								1	0.04
ESR									1

**Table 6:** Spearman’s Rank Correlation (ρ) for Positive 14-3-3***η***.

	14-3-3*η*	Age	WBC	Hgb	Hct	Plt	CRP	C3	ESR
14-3-3***η***	1	−0.06 (*p* = 0.7)	−0.34 (0.09)	0.36 (0.07)	0.22 (0.3)	−0.31 (0.1)	0.27 (0.07)	0.33 (0.3)	−0.12 (0.4)
Age		1	−0.45	0.28	0.36	−0.68	−0.37	−0.06	0.01
WBC			1	−0.55	−0.43	0.62	0.01	NA	0.38
Hgb				1	0.86	−0.54	−0.14	NA	−0.49
Hct					1	−0.53	−0.25	NA	−0.34
Plt						1	0.15	NA	0.56
CRP							1	0.39	0.30
C3								1	−0.18
ESR									1

WBC = white blood cell count, Hgb = hemoglobin, Hct = hematocrit, Plt = platelets, CRP = C-reactive protein, C3 = complement C3, ESR = erythrocyte sedimentation rate, NA = not available.

## Data Availability

The data presented in this study are available in the article and supplementary materials.

## Supplementary Materials

The following are available online at https://www.mdpi.com/2036-7503/13/1/8/s1.
