# Supplementary material for: 14-3-3η Protein as a Potential Biomarker in Juvenile Idiopathic Arthritis
Source: Pediatr Rep. 2021 Jan 25;13(1):65–71. doi: 10.3390/pediatric13010008 (PMC7844622; doi:10.3390/pediatric13010008)
Supplement: Supplementary file 1 [file pediatrrep-13-00008-s001.zip › Dalrymple 14-3-3 supplemental material 2.docx]

Data & Analysis Re: Age & RF, 14-3-3

Counts

  Age grp RF-Neg RF-Pos

    (0,9]      12       5

   (9,14]     12       4

  (14,18]     8       4

  (18,35]     7       6

Proportions

  Age grp     RF-Neg     RF-Pos

    (0,9] 0.7058824 0.2941176

   (9,14] 0.7500000 0.2500000

  (14,18] 0.6666667 0.3333333

  (18,35] 0.5384615 0.4615385

Cochran-Armitage test for trend

Age vs. RF

 
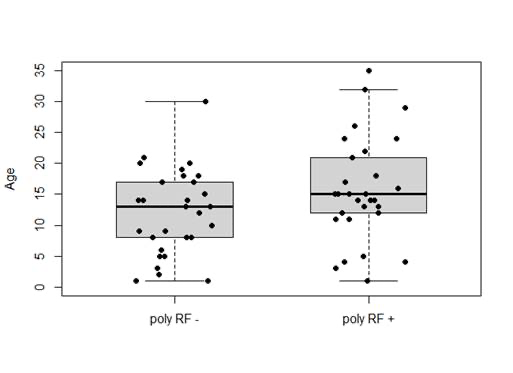


Welch Two Sample t-test

data:  Age by Type.Clean

t = -1.7826, df = 54.014, p-value = 0.08028

alternative hypothesis: true difference in means is not equal to 0

95 percent confidence interval:

-7.6929401  0.4515607

sample estimates:

mean in group poly RF - mean in group poly RF +

               12.06897                15.68966

RF+ subjects tend to be older

Age, RF, & 14-3-3:

 
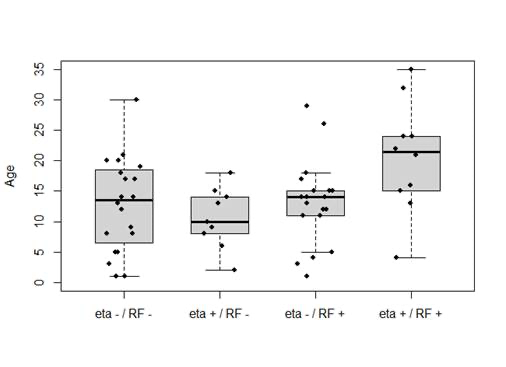


The ANOVA suggests that there are age differences among the eta / RF groups (p = 0.02)

Analysis of Variance Table

Response: Age

           Df  Sum Sq Mean Sq F value  Pr(>F)

eta.rf.grp  3  587.99  196.00  3.5851 0.01946 *

Residuals  54 2952.16   54.67

---

Signif. codes:  0 ‘***’ 0.001 ‘**’ 0.01 ‘*’ 0.05 ‘.’ 0.1 ‘ ’ 1

A multiple comparison analysis (Tukey’s Honest Significant Difference) suggests that the eta + /RF + subjects are on average older than the eta - / RF – subjects (~ 8 years, p = 0.04)

and the eta + / RF – subjects (10 years, p = 0.02) and the eta - / RF + subjects (7.5 years, p = 0.06).

 Simultaneous Tests for General Linear Hypotheses

Multiple Comparisons of Means: Tukey Contrasts

Fit: aov(formula = lm1)

Linear Hypotheses:

                                 Estimate Std. Error t value Pr(>|t|)

eta + / RF - - eta - / RF - == 0  -2.1944     2.9678  -0.739   0.8792

eta - / RF + - eta - / RF - == 0   0.3553     2.3687   0.150   0.9988

eta + / RF + - eta - / RF - == 0   7.8500     2.8636   2.741   0.0393 *

eta - / RF + - eta + / RF - == 0   2.5497     2.9919   0.852   0.8271

eta + / RF + - eta + / RF - == 0  10.0444     3.3973   2.957   0.0230 *

eta + / RF + - eta - / RF + == 0   7.4947     2.8887   2.595   0.0562 .

---

Signif. codes:  0 ‘***’ 0.001 ‘**’ 0.01 ‘*’ 0.05 ‘.’ 0.1 ‘ ’ 1

(Adjusted p values reported -- single-step method)

 
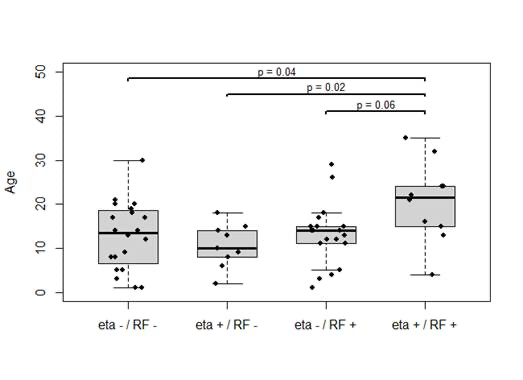


Age associations of the 14-3-3 eta – pos subjects, with continuous 14-3-3 eta response:

 
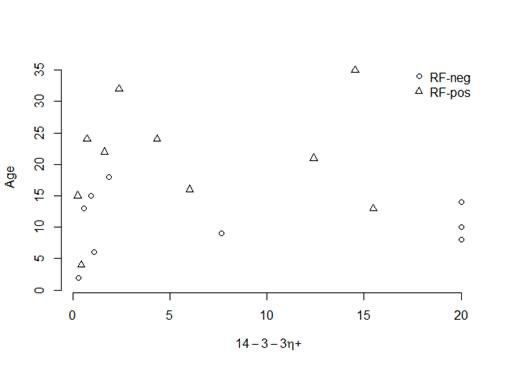


This plot shows that the RF+ subjects tend to be older than the RF- subjects, regardless of 14-3-3 eta concentration. A linear model formalizes this observation:

RF+ subjects are about 10 years older than RF- subjects (p = 0.01), whereas 14-3-3 eta is not associated with age when controlling for RF status (p = 0.6)

Call:

lm(formula = Age ~ X14.3.3eta.trunc + Type.Clean)

Residuals:

     Min       1Q   Median       3Q      Max

-15.9941  -4.2600  -0.3397   3.7691  13.4198

Coefficients:

                    Estimate Std. Error t value Pr(>|t|)

(Intercept)           9.6517     3.1934   3.022  0.00809 **

X14.3.3eta.trunc      0.1123     0.2399   0.468  0.64593

Type.Cleanpoly RF +  10.2944     3.5469   2.902  0.01039 *

---

Signif. codes:  0 ‘***’ 0.001 ‘**’ 0.01 ‘*’ 0.05 ‘.’ 0.1 ‘ ’ 1

Residual standard error: 7.632 on 16 degrees of freedom

Multiple R-squared:  0.3449,   Adjusted R-squared:  0.263

F-statistic: 4.212 on 2 and 16 DF,  p-value: 0.03391
